# Validation of Quality-of-Life assessment tool for Ethiopian old age people

**DOI:** 10.12688/f1000research.130379.2

**Published:** 2024-03-28

**Authors:** Ahmed Muhye, Netsanet Fentahun

**Affiliations:** 1 School of Public Health, College of Medicine and Health Sciences, Bahir Dar University, Bahir Dar, Ethiopia; 2 Department of Nutrition and Dietetics, School of Public Health, College of Medicine and Health Sciences, Bahir Dar University, Bahir Dar, Ethiopia

**Keywords:** Quality of life, WHOQOL-OLD, Validity, Reliability, Old age people, Ethiopia

## Abstract

**Background:**

A valid and reliable quality of life (QOL) assessment tool is critical for identifying health issues, evaluating health interventions, and establishing the best health policies and care plans. One of the tools for this goal is the World Health Organization's Quality of Life Old module (WHOQOL-OLD). It is validated and available in more than 20 languages globally, except Amharic (the widely spoken language in Ethiopia). As a result, the purpose of this study was to translate it into Amharic language and validate it among the elderly people in Bahir Dar City, Northwestern Ethiopia.

**Methods:**

This was a cross-sectional study conducted among 180 community-dwelling old age people in Bahir Dar City, Ethiopia, from January 16 to March 13, 2021. Psychometric validation was achieved through Cronbach’s alpha of the internal consistency reliability test and construct validity from confirmatory factor analysis.

**Results:**

The study participants were aged between 60 and 90 years, with a mean age of 69.44. Females made up 61.7% of the study population, and 40% of them could not read or write. The results showed a relatively low level of quality of life, with a total transformed score of 58.58±23.15. The Amharic version of the WHOQOL-OLD showed a Cronbach’s Alpha value of 0.96 and corrected item-total correlations of more than 0.74. The confirmatory factor analysis confirmed the six-domain model with a chi-square (X2) of 341.98 and a p-value less than 0.001. The comparative fit index (CFI) was 0.98, Tucker-Lewis’s index (TCL) was 0.97, and the root mean square error of approximation (RMSEA) was 0.046.

**Conclusion:**

The Amharic version of the WHOQOL-OLD indicated good internal consistency reliability and construct validity. The tool can be utilized to provide care to Ethiopian community-dwelling old age people.

**Abbreviations**
AVE: average variance extracted
CR: composite reliability
QOL: quality of life
WHOQOL-OLD: world health organization quality of life-for older adults

## Introduction

Advancement in public health sector along with changes in clinical interventions have resulted in a rise in life expectancy in almost every area of the world. ^1^ People are living longer around the world, but they are not necessarily healthier. ^2^^,^^3^ At the same time, the number of years spent living with impairments and chronic illnesses is increasing. The health of old age people is changing more frequently and faster as they live longer lives, which affects their quality of life. ^4^^,^^5^

The World Health Organization (WHO) described QOL as “individuals’ perception of their position in life in the context of the culture and value systems in which they live and in relation to their goals, expectations, standards, and concerns”. ^6^ However, assessing and improving QOL in old age are a difficult undertaking. This is related to the complicated concept of QOL, the identification of many instruments, and the subjectivity of how older people and healthcare practitioners judge their patients' health. ^7^^–^^9^ Despite this, if old age people have their independence, autonomy, and good physical health, as well as remain active, find purpose in their lives, and fulfill their social obligations, their QOL may be good or at least maintained. ^10^^–^^12^

Furthermore, WHOQOL-OLD has been developed specifically for measuring QOL in old age people ^13^ and its novel form contains a total of 24 items assembled into six domains, each with four items: autonomy (AUT), past, present, and future activities (PPF), sensory abilities (SAB), social participation (SOP), death and dying (DAD), and intimacy (INT). ^14^

Researchers have identified significant disparities in the quality of life experienced by older adults residing in developed and developing countries. ^9^ In a comprehensive study encompassing several developing nations, including China, India, Ghana, South Africa, Mexico, and Russia, investigators examined the concept of QoL by soliciting respondents' assessments of their satisfaction levels pertaining to various life domains, employing a 5-point rating scale. The results uncovered substantial variations in overall QoL scores across these countries, ranging from 45.5 (Ghana) to 52.1 (China). ^13^ Furthermore, noteworthy differences were observed based on socio-economic factors. Notably, in high-income countries, sensory abilities and intimacy emerged as the QOL sub-scale with the highest scores, ^14^^–^^16^ whereas in low-income countries, social participation (SOP) obtained the highest QOL sub-scale score. ^17^^,^^18^ These disparities underscore the distinctive challenges encountered by aging populations residing in contexts with limited resources. Consequently, these findings assume significance in informing policies and interventions aimed at augmenting the well-being of older adults. ^9^

In contrast to their counterparts in developed nations, older adults in many developing countries confront obstacles due to insufficient healthcare infrastructure and underdeveloped geriatric systems. The accessibility of quality healthcare remains a formidable challenge for this demographic. Simultaneously, social security systems in developing countries frequently fail to furnish adequate support to older adults. A considerable number of older individuals lack access to pensions, retirement benefits, or social safety nets. Facilitating meaningful change necessitates active engagement with local communities, policymakers, and older adults themselves. Prioritizing preventive healthcare, early detection and management of chronic conditions, promotion of healthy behaviors, provision of accessible and comprehensive healthcare services, and addressing social determinants of health are all imperative for enhancing the quality of life for older adults. Additionally, adopting a holistic approach that encompasses physical, mental, and social well-being assumes paramount importance in elevating the overall quality of life for this aging population, which experiences increased longevity. ^9^^,^^13^

Available studies in Ethiopia did not use the WHOQOL-OLD. They instead used other tools, such as the medication-related quality of life (MRQoL), ^20^ Control, Autonomy, and Self-realization (CASP), ^21^ and the World Health Organization Quality of Life-brief version (WHOQOL-BREF). ^22^^,^^23^ To the best of our knowledge, these tools were neither developed for nor have yet been rigorously validated for Ethiopian old age people. Still, the accessible tools for evaluating QOL are usually designed and validated in developed nations, which have distinct cultural, socio-economic, and life standards contrary to those of African nations. Furthermore, the majority of old age Africans are illiterate, making it difficult to use QOL questionnaires that demand users to read and write. ^24^

The lack of validated instruments troubles the accuracy of the data generated and its extrapolation to a larger population, as well as the ability to compare findings through studies. Subsequently, low-quality data can have a detrimental impact on policies and services, as well as efficient use of resources. ^25^ Therefore, this study aimed to translate and validate the WHOQOL-OLD tool for Ethiopian old age people.

## Methods and Materials

### Study setting

This study was conducted in Bahir Dar City, the capital of the Amhara Regional State. Bahir Dar is located in Amhara Regional State, Northwest Ethiopia, which is 565 kilometers away from Addis Ababa, the capital city of Ethiopia.

### Study design and period

A cross-sectional study design was conducted from January 16 to March 13, 2021.

### Study population, sample size and sampling procedures

This study utilized two groups of the population. The first group were health care experts used for content validation, and the second group were community-dwelling old age people for psychometric validation. For the expert judgment, 10 healthcare experts were purposefully selected based on the guideline recommendation for the Delphi technique. ^26^ For the psychometric validation, a participant-to-variables ratio of 10:1 was followed as a rule of thumb. ^27^ Since the mini nutritional assessment tool has 18 items, a minimum of 180 study participants were selected, and the study population was used for this WHOQOL-OLD tool validity study too. Community-dwelling old age people selected in multistage cluster sampling from Belay Zeleke, one of the sub-cities of Bahir Dar City, Northwest Ethiopia were used for this study. Community-dwelling people age 60 years and above, living in the city administration at least for six months, being capable of describing their lived experience, and being able to understand and speak the local Amharic language were included. While those who had significant spine curvature (scoliosis or kyphosis) and had both extremities amputated were excluded. The detailed study methods for study population, sample size, and sampling procedures were described in the previous study. ^28^

### Validation process

This tool validation study was conducted in three stepwise phases. The first phase was to review existing QOL assessment tools for old age people. In the second phase, selection, translation, and review of the tool by experts were conducted. In the last phase, psychometric validation among community-dwelling old age people was performed.

### Review of Existing Quality of Life Assessment Tools

Quality of life (QOL) has been conceived and assessed in a variety of ways based on the paradigm, discipline, target community, and time frame of the study investigating it. ^29^ Around the world, numerous tools have been established for measuring QOL in adults and validated for the elderly. ^9^^,^^30^ Only in Africa, 14 unique tools were identified from 22 studies to measure QOL in old age people. ^24^ Furthermore, instruments have been developed specifically for measuring QOL in old age people, including the WHOQOL-OLD, ^13^ the Elderly Quality of Life Index (EQLI), ^31^ the Older People’s Quality of Life (OPQOL) questionnaire, ^32^ and the World Health Organization Quality of Life-AGE questionnaire (WHOQOL-AGE). ^33^

The WHOQOL-OLD novel form contains a total of 24 items assembled into six domains, each with four items: autonomy (AUT), past, present, and future activities (PPF), sensory abilities (SAB), social participation (SOP), death and dying (DAD), and intimacy (INT). The module evaluates mostly the two-week duration of testing in self-report or interviewer-administered form. Although each object is rated on a Likert scale of 1 to 5, they differ in their anchors. Each domain provides an individual score ranging from 4 to 20. The component values can also be converted to a scale of 0 to 100. Furthermore, summing the individual item values yields total scores from 24 to 120, with higher scores indicating better QOL. ^14^

### Translation and Cultural Adaptation of WHOQOL-OLD

The WHOQOL-OLD instrument was chosen from the available QOL measurement tools to translate and culturally adapt for the context of our community because it: (1) is designed specifically for elderly people; ^13^ (2) is the most comprehensive multidimensional instrument that covers multiple components of QoL; ^13^^,^^14^^,^^34^ (3) contains items that are particularly relevant for old age people and are absent from the other instruments, such as autonomy, intimacy, and death and dying; ^13^ (4) is subjective and culturally sensitive; ^35^^,^^36^ (5) showed good reliability and validity in the assessment of QOL for older participants with multi-language versions; ^37^^,^^38^ and (6) is freely available for research use. ^14^

**Figure 1.**
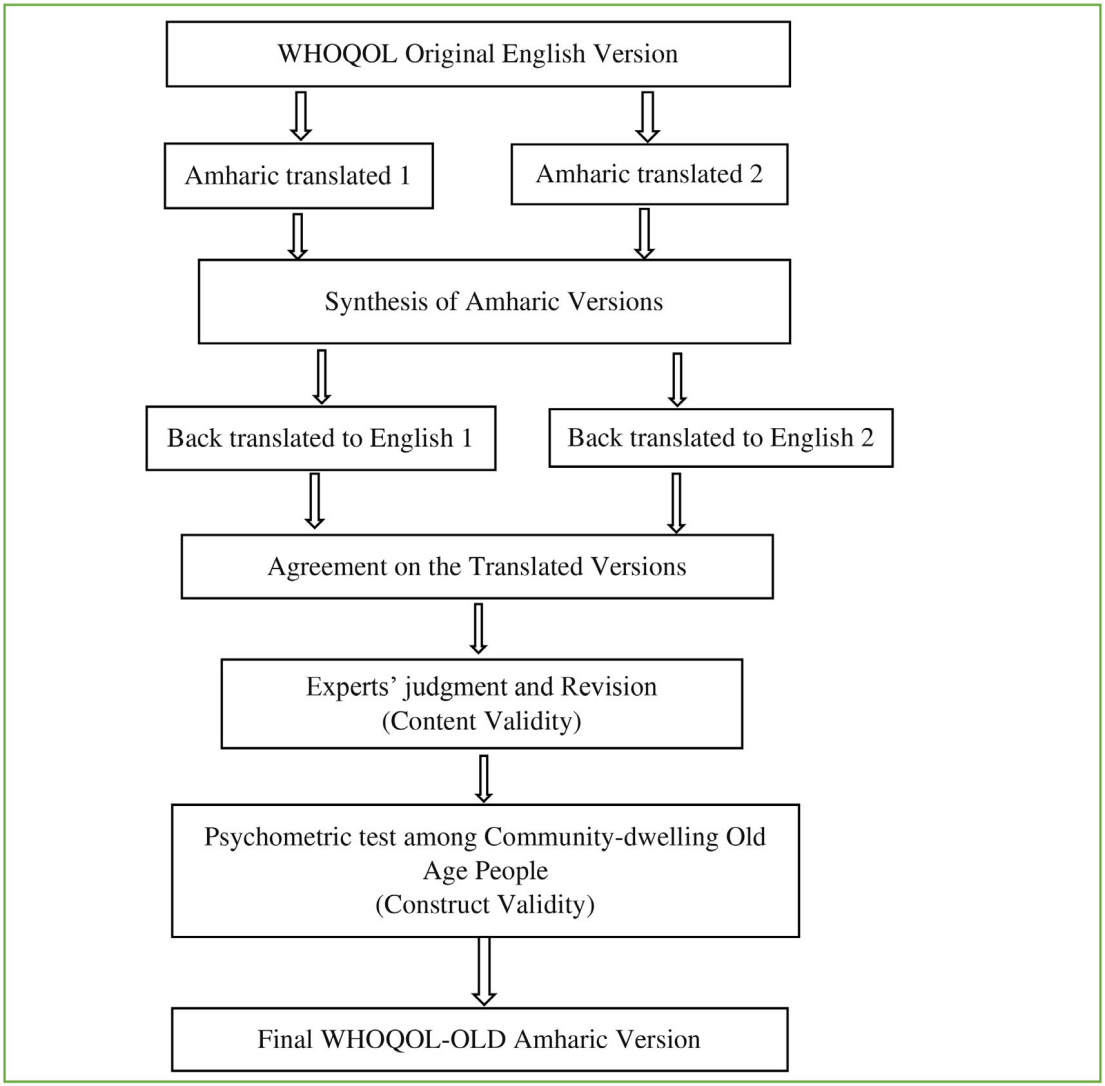
Validation process of the Amharic version of WHOQOL-OLD, Bahir Dar City, 2021.

The English version of the WHOQOL-OLD questionnaire was initially translated into the Amharic local mother tongue version independently by bilingual internists and human nutritionists trained at master’s degree level. These two translators were selected respectively as they are experienced in care providing for old age people and nutrition research and might be familiar with the intent of each item and/or the tool as a whole. The two Amharic versions were then combined, and any inconsistencies were settled by consensus. The translated Amharic version was next translated back into the original English language to ensure the accuracy of the translation. This was done again by two independent bilingual, native Amharic-speaking language translators trained at masters’ degree level. Finally, the experts’ group reviewed both versions of the translations and reached a conclusion on all items to get a final version of the translated questionnaires ( Figure 1).

### Data collection

Data were collected from two groups: healthcare experts and community-dwelling old age people, in exploratory mixed qualitative and quantitative methods. Each expert evaluated the content validity of the tool through face-to-face contact. The experts and old age people’s comments were used for words, grammar, clarity, appropriate scoring and applicability of items. After incorporating the experts’ comments, psychometric validation was conducted among community-dwelling old age people.

Six urban health extension workers and six bachelor of science nurses collected the data after two days of training. The principal investigator and a master’s degree trained nutritionist supervised the data collection process. The data were collected through face-to-face interviews using the standardized Amharic version of the questionnaires. Assistance from family members or caregivers was also used.

### Data Analysis

The international business machines corporation statistical package for the social science (IBM SPSS) version 23 ^39^ (RRID:SCR_002865, URL: http://www-01.ibm.com/software/uk/analytics/spss/) and the extension of Analysis of Moment Structures (AMOS) via the maximum likelihood estimation method ^40^ were used to analyzed the data. Socio-demographic characteristics of the study participants were expressed in descriptive statistics. Whereas, the statistical analysis of the WHOQOL-OLD tool in this study was done in stages. The values for all negatively phrased items coded with a number of 1, 2, 6, 7, 8, 9, and 10 on the tool were first reverse-scaled to match the values for positively phrased questions. Second, the statistic assumptions of normality and outliers were verified. Using the squared Mahalanobis distance (d ^2^) greater than 0.05 for each item, ^40^ no more severe multivariate outliers were discovered, and none were deleted. Furthermore, normalized kurtosis values and critical ratios of less than 5.00 indicated that the data were normally distributed. ^40^ Thirdly, total and mean scores were computed for each domain. Finally, the overall total score was translated into a score with a range of 0 to 100.

### Validity measurement

**Content validity and acceptability**

To assess the acceptability of the Amharic version of the WHOQOL-OLD, the response rate and floor and ceiling effects of summary scores were examined. If more than 15% of respondents received the lowest bad health score or the highest good health score possible score, there were floor and ceiling effects. ^41^

**Construct Validity**

Exploratory and confirmatory factor analyses were performed, respectively, to check construct validity. The principal component analysis (PCA) with Promax rotation was performed to evaluate the sample adequacy and check whether the items in the translated questionnaires were organized comparably to the novel questionnaires. Oblique rotation was used rather than orthogonal since we expected that the factors of the tool would be intercorrelated, as previously verified by other studies. ^17^^,^^42^ The Kaiser-Meyer-Olkin (KMO) test at a minimum level of 0.60 was used to determine whether the items were sufficiently correlated to allow for factor analysis. ^43^ Whereas, Bartlett's test of sphericity with a *p-value* less than 0.05 was used to examine the inter-correlations between items. In addition, the eigenvalues of more than one rule and a graphic review of the scree plot were employed to decide the number of factors to maintain. Items had to be related to a single component, and each rotated component had to have at least four items to assess component affiliation. The proportion of explained variance of more than 60% was used to measure the factors' ability to describe the data. ^43^

The data were then exported to AMOS version 23 for confirmatory factor analysis (CFA). ^40^ A predefined six-factor model in first and second-order CFA was used to test the construct validity of the Amharic version of the WHOQOL-OLD tool. The first model is a congeneric measuring model that depicts the six-factor structure in which each item on the questionnaire was linked to the underlying latent construct of its predicted aspect. The second-order factor was introduced to see if the construct “QOL” could be represented by a single dimension. ^43^

At least one test from each of the four typical model fit indexes was used for the acceptability of CFA suggested variables. These included the chi-squared test (X ^2^) from the overall model fit, the goodness-of-fit index (GFI), the root mean square error of approximation (RMSEA), or the standardized root mean square residual (SRMR) from the absolute fit indexes; and the comparative fit index (CFI), the normed fit index (NFI), non-normed fit index (NNFI), or Tucker-Lewis index (TLI) from the relative or incremental fit index; and the Akaike Information Criteria (AIC) or Bayesian Information Criteria (BIC) from the predictive fit indicators. ^44^^–^^46^ The recommended model is usually the one with the least AIC and BIC statistic value ^46^ and an RMSEA of less than 0.08. ^44^^,^^45^ While the GFI, CFI, NFI, and NNFI scores more than 0.90, especially those near one, indicated good fitness. ^44^^,^^45^

The CFA also took into account for both convergent and divergent validity. Convergent validity was evaluated using the factor loading, AVE, and composite reliability (CR) tests. Good convergent validity was considered if the total correlations and factor loading or inter-item correction values exceeded 0.50 and 0.30, respectively. ^43^

The AVE and composite reliability (CR) values were calculated as:

$$\mathrm{AVE}=\frac{\sum_{i=1}^{n}\;{\mathrm{Li}}^{2}}{n}\;\mathrm{CR}=\frac{{\left(\sum_{i=1}^{n}\;\mathrm{Li}\right)}^{2}}{{\left(\sum_{i=1}^{n}\;\mathrm{Li}\right)}^{2}+\sum_{i=1}^{n}\;\mathrm{ei}}$$

Where, L _i_ is the factor loading for i ^th^ construct n is the number of item indicators for a construct and e _i_ is the error variance term for a construct.

The values of AVE of 0.5 or more and composite reliability (CR) of 0.7 or higher were used to see if the items logged under each facet/domain were estimating the same concept. ^43^

The divergent or discriminant validity of the Amharic version of the WHOQOL-OLD construct was achieved when the coefficient of cross-loading (correlation among the components) did not exceed 0.85. ^43^ Additionally, the value of maximum shared variance (MSV) being less than the value of AVE was used as an indication of divergent validity. ^43^

**Reliability**

Cronbach's alpha (α) was used to measure internal consistency, and a value greater than 0.7 was taken as a benchmark. ^47^ In addition, construct reliability (CR) based on the factor loading after CFA and a coefficient of more than 0.70 was considered satisfactory. ^43^ Furthermore, the Pearson correlation coefficient was used to correct the reliability coefficient for the 24 items of the Amharic version of the WHOQOL-OLD scale.

**Data quality control**

Data collection questionnaires were adapted from previously validated standards. The data collectors and supervisors took two days of training on the study’s purpose and the utilization of data collection tools. Statistical data assumptions were checked following the prescribed processes.

## Results

### Sociodemographic characteristics of study participants

A total of 180 community-dwelling old age people aged from 60 to 90 years participated in this study. The mean age was 69.44, with a standard deviation of 6.8. The majority of the study participants were females (61.7%) and orthodox religious followers (73.9%). More than half (53.3%) of the respondents were married and lived with their spouses, and 40% of them could not read and write ( Table 1).

**Table 1.** Sociodemographic characteristics of the study participants in Bahir Dar City, 2021.

S/No	Respondents’ characteristics		Frequency	Percentage
1.	Sex	Female	111	61.7
		Male	69	38.3
2.	Age	60—64	40	22.2
		65—69	56	31.1
		70—74	42	23.3
		75—79	23	12.8
		80—84	11	6.1
		≥85	8	4.4
3.	Religion	Orthodox	133	73.9
		Islam	45	25
		Protestant	2	1.1
4.	Marital status	Single	5	2.8
		Married	96	53.3
		Divorced	5	2.8
		Widowed	74	41.1
5.	Educational status	Cannot read and write	72	40.0
		Can read and write	45	25.0
		Primary education	35	19.4
		Secondary education	16	8.9
		Certificate and above	12	6.7
6.	Occupation	House wife	75	41.7
		Daily-laborer	7	3.9
		Merchant	25	13.9
		Pension	61	33.9
		No work	12	6.7
7.	Lived with	Spouse	96	53.3
		Children	59	32.8
		Alone	21	11.7
		Other persons	4	2.3

### Validity of the Amharic version of the WHOQOL-OLD Tool

**Content validity and acceptability**

As experts reviewed, every item in the tool was socially acceptable and had no sensitive words. Minor changes, such as word and phrase expansion and substitution of more relevant Amharic terminology and phrases were made to make the items clear and more accurate. Moreover, there were no major difficulties encountered throughout the data collection period, and the scale was completed on each participant in 25 to 35 minutes. The result showed a 100% response rate without missing any item. No significant concern was raised in their remarks about the understandability of the questions and response items. The ceiling and floor effects of each domain in the Amharic version of WHOQOL-OLD varied from 1.8 to 7.7% and 0 to 2.9 %, respectively.

**Construct validity**

All variables of the tool were correlated with more than 0.306 in the matrix correlation, satisfying the requirement of the presence of two or more correlated variables with more than a 0.30 coefficient. In addition, the measure of sampling adequacy, located on the diagonal of the anti-image correlation matrix of SPSS, was greater than 0.80 for each variable in the first iteration. This is commendable and does not necessitate the removal of any items. Furthermore, the Kaiser-Meyer-Olkin (KMO) measure of sampling adequacy was 0.943, and Bartlett’s test of sphericity was statistically significant (X ^2^ = 3,915.790; n = 180; *df* = 276; P<0.0001). These indicate that all the 24 variables that remained in the analysis satisfied the criteria for appropriateness of factor analysis.

In the same way, the 24 variables appeared to measure five underlying components using the latent root criterion, commonly known as the Kaiser criterion (eigenvalues greater than 1.0). These variables are responsible for 78.2% of the total variance explained. The results were identical when a fixed six component based on prior knowledge was used. While the scree plot suggested that six factors would be appropriate when considering the changes in eigenvalues. Moreover, the communality value was satisfactory for all variables, with a minimum value of 0.687. Since each variable has more than 0.50, there is no need for communality variable removal.

With four items on each component, all of the 24-items are heavily factor loaded with more than 0.5. Explicitly, Factor 1 was loaded with the four items of PPF activities. The four autonomy (AUT) components, on the other hand, were loaded onto Factor 2. The loadings of the factors ranged from 0.649 to 0.846. Furthermore, there were no instances of cross-loading between the components.

**Figure 2.**
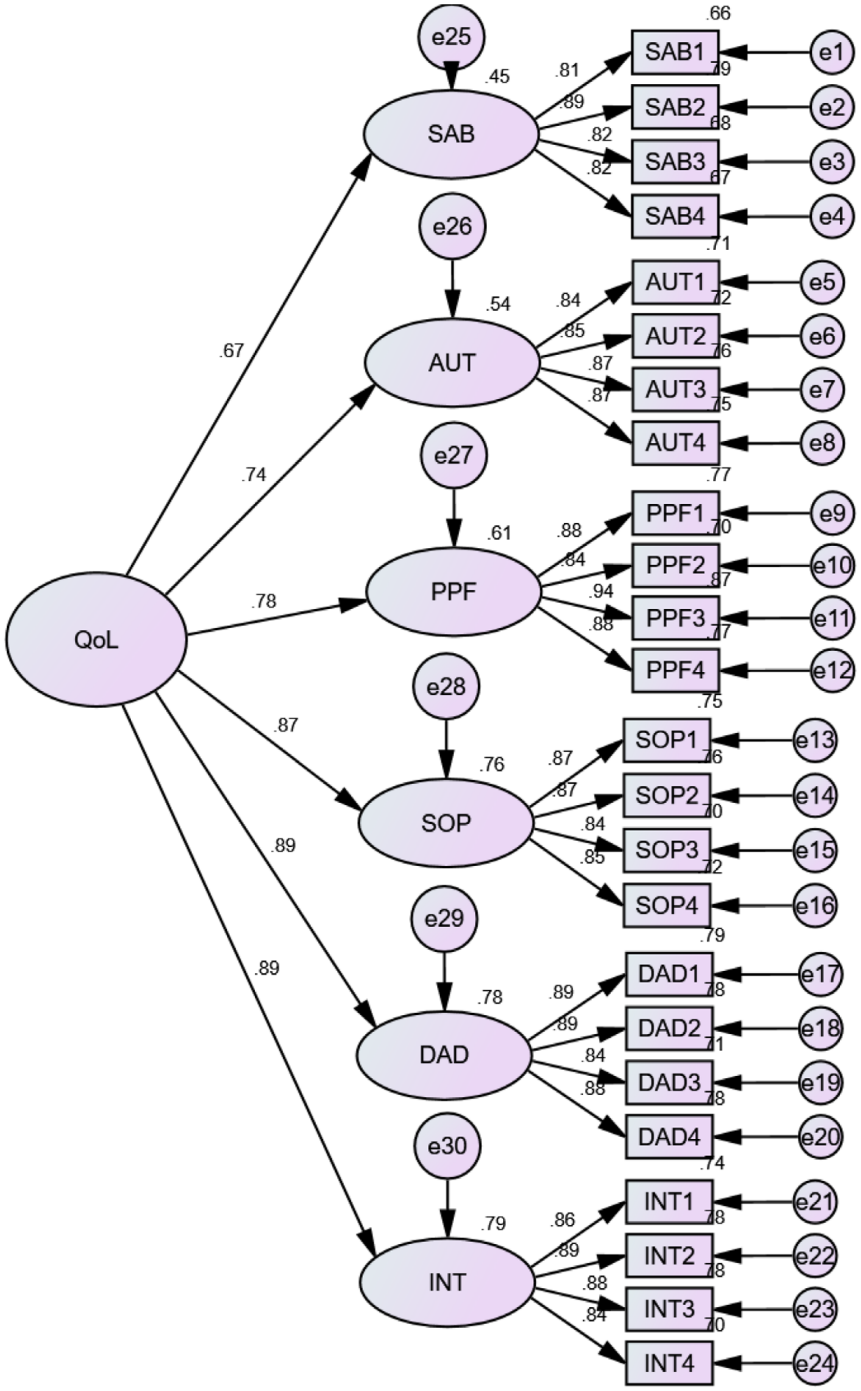
Second-order confirmatory factor analysis of the Amharic version of WHOQOL-OLD among community-dwelling old age people in Bahir Dar City, 2021.

The extended analysis of EFA; CFA) was used to see if the results fit a postulated measurement model. The results of the first-and second-order CFA showed that all WHOQOL-OLD facets are adequately represented on the linked items by substantial standardized loadings above 0.5 ( Figure 2). When the goodness of fit index parameters of both models was compared using standard structural equation modelling (SEM) procedures, it was clear that adding the second-order common component did no influence on the model fit. All four indices displayed an acceptable fit, except the value of the goodness-of-fit index (GFI) and adjusted goodness-of-fit index (AGFI), which are slightly below 0.90 ( Table 2).

**Table 2.** Model fit statistics of the first and second order of CFA for the Amharic version of WHOQOL-OLD domains, Bahir Dar City, 2021.

S/No	Goodness of fit indices	Parameter category	First-order factor model	Second-order factor model
1.	Overall fit	Chi-square ( *X ^2^*)	326.308	341.982
		Degree of freedom ( *df*)	237	246
		Relative likelihood ratio ( *X ^2^/df*)	1.377	1.390
2.	Absolute fit	p-value	<0.0001	<0.0001
		Goodness-of-fit index (GFI)	0.873	0.867
		Root mean square error of approximation (RMSEA) (90% CI)	0.046 (0.033-0.058)	0.047 (0.034-0.058)
		Standardized root means square residual (SRMR) (≤0.08)	0.0448	0.0393
3.	Incremental fit	Adjusted Goodness-of-fit index (AGFI)	0.839	0.838
		Normed fit index (NFI)	0.921	0.917
		Comparative fit index (CFI)	0.977	0.975
		Relative fit index (RFI)	0.908	0.907
		Tucker-Lewis index (TLI)	0.973	0.972
4.	Predictive fit	Akaike information criteria (AIC)	452.308	449.982
		Bayesian information criteria (BIC)	653.465	622.401

The CFA also took into account both convergent and divergent validity. The scaling analysis revealed that almost all of the items had good correlations with their respective sub-scales (r≥0.65), indicating that the instrument has strong convergent validity. Additionally, the findings confirmed that all item loadings on their own factor were greater than 0.800, which is required for convergent validity.

Furthermore, AVE and composite reliability (CR) values for each construct of the Amharic version of WHOQOL-OLD were more than 0.5 and 0.7, respectively. The values for the total score of the tool were respectively 0.68 and 0.92, which are more than the acceptable range. The AVE estimations ranged from 69.8% for SAB to 77.6% for PPF activities, respectively. Thus, all constructs exceed the 50% rule of thumb, which states that items measuring similar restrictions are loaded into one domain. The AVE values are also larger than the MSV values, which is important for divergent or discriminant validity. Additionally, the calculated correlation coefficient between all six components of the model in IBM-SPSS-AMOS does not exceed 0.85. As a result, we conclude that the measuring tool for the construction of the Amharic version of WHOQOL-OLD has attained divergent or discriminant validity ( Table 3).

**Table 3.** Estimates of average variance extracted, composite reliability, and maximum shared variance of Amharic version of WHOQOL-OLD, Bahir Dar City, 2021.

Construct	Average variance extracted (AVE)	Construct reliability (CR)	Maximum shared variance (MSV)
Sensory abilities (SAB)	0.698	0.902	0.287
Autonomy (AUT)	0.723	0.912	0.358
Past, present, and future activities (PPF)	0.776	0.933	0.453
Social participation (SOP)	0.740	0.919	0.554
Death and dying (DAD)	0.757	0.926	0.554
Intimacy (INT)	0.753	0.924	0.554
Total score of QOL	0.678	0.919	

**Reliability**

The Cronbach’s Alpha (α) values of the Amharic version of WHOQOL-OLD were above 0.90, varying from 0.902 for SAB to 0.932 for PPF activities. The total scale has a Cronbach’s alpha (α) value of 0.963. Meanwhile, Cronbach’s alpha coefficient of each domain as well as the total scale did not increase when each item was deleted, indicating that all had constructive contributions to their facets as well as the total scale ( Table 4).

**Table 4.** Cronbach’s alpha and Item-total statistics of each domain of Amharic version of WHOQOL-OLD, Bahir Dar City, 2021.

WHOQOL-OLD domain/facet	Item No	Item text	Cronbach's Alpha (α)	Corrected Item-Total Correlation	Cronbach's Alpha if Item Deleted
1. Sensory abilities (SAB)	01.	Sensory impairment affecting daily life	0.902	0.780	0.876
	02.	Loss of sensory abilities affects participation in activities		0.826	0.857
	10.	Problems with sensory functioning affect social interaction		0.783	0.874
	20.	Rate sensory functioning		0.742	0.887
2. Autonomy (AUT)	03.	Freedom to make own decisions	0.915	0.786	0.896
	04.	Feeling in control of your future		0.796	0.892
	05.	People around you are respectful of your freedom		0.830	0.881
	11.	Able to do things you would like to do		0.817	0.887
3. Past, present and future activities (PPF)	12.	Satisfied with opportunities to continue achieving goals	0.932	0.836	0.913
	13.	Received the recognition you deserve in life		0.801	0.925
	15.	Satisfied with what you have achieved in life		0.894	0.894
	19.	Happy with things to look forward to		0.839	0.913
4. Social participation (SOP)	14.	Have enough activities to perform each day	0.916	0.808	0.891
	16.	Satisfied with the way you use your time		0.822	0.885
	17.	Satisfied with the activity level		0.787	0.898
	18.	Satisfied with the opportunities to participate in community activities		0.813	0.889
5. Death and dying (DAD)	06.	Concerned with the way you will die	0.928	0.843	0.903
	07.	Afraid of not being able to control death		0.853	0.900
	08.	Scared of dying		0.804	0.916
	09.	Fear pain before death		0.832	0.907
6. Intimacy (INT)	21.	Feel a sense of companionship in life	0.923	0.827	0.899
	22.	Experience love in life		0.830	0.897
	23.	Opportunities to love		0.847	0.892
	24.	Opportunities to be loved		0.786	0.913

In addition, the Pearson correlation revealed high correlation coefficients between items and their theorized domains (inter-item relations) and the six domains themselves as well ( Table 5).

**Table 5.** Correlation coefficients between items and domains or a total score for the Amharic version of WHOQOL-OLD in Bahir Dar City, 2021.

Item number	Sensory abilities (SAB)	Autonomy (AUT)	Past, present, and future activities (PPF)	Social Participation (SOP)	Death and dying (DAD)	Intimacy (INT)	Total score
01.	***0.872***	0.421	0.361	0.405	0.436	0.400	0.582
02.	***0.909***	0.454	0.493	0.505	0.464	0.494	0.671
10.	***0.885***	0.502	0.373	0.405	0.441	0.426	0.609
20.	***0.854***	0.508	0.491	0.511	0.522	0.537	0.692
03.	0.512	***0.884***	0.550	0.492	0.551	0.592	0.723
04.	0.456	***0.887***	0.529	0.516	0.509	0.539	0.694
05.	0.484	***0.911***	0.491	0.461	0.523	0.494	0.677
11.	0.463	***0.893***	0.533	0.517	0.527	0.514	0.695
12.	0.507	0.562	0.571	0.670	***0.914***	0.687	0.797
13.	0.453	0.530	0.506	0.678	***0.921***	0.653	0.762
15.	0.444	0.519	0.573	0.647	***0.887***	0.670	0.763
19.	0.516	0.531	0.592	0.703	***0.907***	0.691	0.804
14.	0.429	0.513	***0.909***	0.579	0.542	0.643	0.745
16.	0.428	0.503	***0.884***	0.586	0.597	0.609	0.742
17.	0.458	0.544	***0.946***	0.601	0.533	0.632	0.765
18.	0.469	0.588	***0.908***	0.579	0.587	0.572	0.760
06.	0.471	0.508	0.592	***0.897***	0.693	0.646	0.778
07.	0.499	0.472	0.568	***0.903***	0.666	0.626	0.763
08.	0.474	0.496	0.590	***0.879***	0.643	0.632	0.760
09.	0.408	0.506	0.546	***0.895***	0.654	0.591	0.736
21.	0.468	0.532	0.579	0.594	0.643	***0.903***	0.759
22.	0.502	0.549	0.672	0.648	0.675	***0.907***	0.808
23.	0.487	0.515	0.574	0.630	0.666	***0.915***	0.774
24.	0.449	0.564	0.602	0.646	0.698	***0.883***	0.785

In comparison to the other domains, the correlation coefficients between items and their postulated domains were substantially higher. Furthermore, the domains themselves were moderately correlated with each other. The lowest correlation was observed between SAB and PPF activities with a correlation coefficient value of 0.489. The highest correlation was observed between the correlation of SOP and INT with DAD, both with a correlation coefficient value of 0.744. Additionally, all of the domains were highly connected with the total QOL score, with the SAB and INT having the lowest (0.726) and highest (0.867) correlation coefficients with the overall QOL score, respectively ( Table 6).

**Table 6.** Correlation coefficient between domains and total score of Amharic versions of WHOQOL-OLD among community-dwelling old age people in Bahir Dar City, 2021.

Domains/facets	Sensory abilities (SAB)	Autonomy (AUT)	Past, present, and future activities (PPF)	Social Participation (SOP)	Death and dying (DAD)	Intimacy (INT)	Overall score
Sensory abilities (SAB)	1						0.726 ^**^
Autonomy (AUT)	0.536 ^**^	1					0.780 ^**^
Past, present, and future activities (PPF)	0.489 ^**^	0.588 ^**^	1				0.825 ^**^
Social Participation (SOP)	0.519 ^**^	0.554 ^**^	0.643 ^**^	1			0.850 ^**^
Death and dying (DAD)	0.529 ^**^	0.590 ^**^	0.617 ^**^	0.744 ^**^	1		0.861 ^**^
Intimacy (INT)	0.528 ^**^	0.599 ^**^	0.673 ^**^	0.698 ^**^	0.744 ^**^	1	0.867 ^**^

^**^ Values are statistical significance (p<0.001) between facet- facet and total score relation.

## Discussion

This is the first study examination of the psychometric properties of the WHOQOL-OLD for a representative sample of the Ethiopian population aged 60 years and older. The results revealed that all items in the Amharic version of the WHOQOL-OLD were simple to understand and respond to, indicating that the scale is practicable. Similar findings were reported from psychometric studies of Korea ^42^ and Iran. ^48^ In addition, all of the domain scores and the overall score revealed less than 15.0% ceiling and floor effects, which is acceptable for all subscales. ^41^ This classification indicated that the Amharic version of WHOQOL-OLD had no significant floor and ceiling effects, indicating its discriminant ability. This is consistent with the other cultural studies conducted in Korea ^42^ and Iran. ^48^

In terms of content validity, this study yields statistically significant item-facet correlation coefficients that are identical to those found in China. ^37^ Moreover, the results of CFA for a six-factor model indicated acceptable construct validity that best fit the study data and was congruent with the reported priori factor structure of the original scale ^13^^,^^14^ and in the validation studies of Vietnam, ^17^ Korea, ^42^ Iran, ^48^ and the Netherlands. ^15^

Our analysis also revealed the psychometric qualities of the Amharic version of the WHOQOL-OLD, such as RMSEA of 0.047, CFI of 0.975, GFI of 0.867, and NFI of 0.917. These are comparable to, if not better than, those reported in the worldwide WHOQOL-OLD field research ^14^ and those of other country versions in the Netherlands, ^15^ Vietnam, ^17^ Korea, ^42^ and Iran. ^48^

The CFA-based fit indices in this study are also acceptable as measures of divergent validity, which is a subtype of construct validity. ^43^ There was no evidence of scaling error, as the tool’s items discriminate significantly between their own and other domains, demonstrating divergent validity.

Furthermore, all corrected item-total correlations and factor loadings based on the six-factor CFA model appear higher than 0.30, which is consistent with a study from Vietnam. ^17^

Internal consistency Cronbach's alpha value in the current study demonstrated high-reliability coefficients and item-scale respective inter-item correlations for the total and subdomains of the Amharic version of WHOQOL-OLD. The findings are higher than compared to those of prior research conducted in Vietnam, ^17^ Korea, ^42^ and Iran. ^48^ This could be because of socio-cultural differences, with older people residing in different countries. There could also be a chance of reporting bias based on respondents' willingness and ability to provide accurate responses, especially when it comes to the length of time in the interview.

### Strengths and limitations of the study

To our knowledge, this is the first study that adapt and validate the WHOQOL-OLD tool in Ethiopia. This study has strengths, as the data collection and the validation were conducted both from experts and community-dwelling old age people, which could have decreased some bias. Data collection was conducted by experienced health extension workers and nurses.

Despite these strengths, this research has few limitations. The primary weakness is the self-reported nature of the tool, which can lead to the under-or overrepresentation of results. Second, it was conducted among community-dwelling old age people in urban locations; as a result, the findings may not apply to those living in rural or institutional settings. Third, test-retest reliability and sensitivity to change of the instruments could not be tested due to the study's cross-sectional design.

## Conclusion

The current study found that the translated Amharic versions of the WHOQOL-OLD tool indicated robust internal consistency and construct validity. The instrument can be utilized in routine care provision activities among the community-dwelling old age people in Bahir Dar, Northwestern Ethiopia. Other social care-providing organizations can also use the Amharic version of WHOQOL-OLD to estimate the impacts of their policies, services, or targeted interventions might have on elder people. However, since Ethiopia is a country of socio-cultural diversity, more research on multiethnic and multi-cultural issues is required.

## Ethical approval

This research was conducted as part of a Ph.D. dissertation that received ethical approval from Bahir Dar University (R.N./IRB/003/2021). In addition, participantion was entirely voluntary, and every participant gave informed consent.

## Authors’ contribution

Muhye Ahmed planned the research, analyzed the data, and wrote the paper. Fentahun Netsanet was involved in the design, data analysis, manuscript preparation, and critical evaluation of the study. Both authors read and approved the final manuscript.

## Data Availability

Dryad: Data from: Validation of Quality-of-Life assessment tool for Ethiopian old age people. https://doi.org/10.5061/dryad.zkh1893dq. ^49^ This project contains the following underlying data:
- Quality of life SPSS data (spss.sav) - README data (MD document) The study questionnaire. ^49^ This project contains the following extended data:
- The study questionnaire ^49^ - STROBE checklist- for quality of life as cross-sectional study. ^49^ Data are available under the terms of licensed under a CC0 1.0 Universal (CC0 1.0) Public Domain Dedication license.
