# Supplementary material for: Validation of Quality-of-Life assessment tool for Ethiopian old age people
Source: F1000Res. 2024 Mar 28;12:282. Originally published 2023 Mar 14. [Version 2] doi: 10.12688/f1000research.130379.2 (PMC11128050; doi:10.12688/f1000research.130379.2)
Supplement: Peer review history

# Reviewer report: Reviewer response for version 2

**DOI:** 10.5256/f1000research.164253.r277783

**Published:** 2024-05-25

**Authors:** Erhan Eser

**Affiliations:** 1 Department of Public Health, Manisa Celal Bayar Universitesi, Manisa, Manisa, Turkey

GENERAL COMMENTS

 The authors aimed to show whether the Amharic version of the scale is a valid and reliable scale for Ethüopian (Amharic ) language speaking patients by presenting the psychometric analysis results of the Turkish version of the WHOQOL-OLD scale.

 The authors followed the steps of cultural adaptation and performed almost complete psychometric analyzes based on field data. This manuscript is a successful work in this context.

 INTRODUCTION

 Adequate

 METHODS

 Some minor points that needs consideration, such as:

1. Normalized kurtosis values were taken into account whereas Skewness was not? I suggest to interpret normal distribution via Skeness evaluation as well.
2. Test re-test approach is lacking for testing the reliability of the scale (in addition to Chronbach)
3. Although is not adequate for psychometric analyses (180 for 24 item scale) the KMO results (>0.5) A KMO value of over 0.5 eliminates this problem.
4. Confirmatory analyses all are comprehensive and the results are satisfactory except for “Kown Groups validity” analyses. What I mean is the comparision of the dimension (and overall) What , I mean comparisons of subcategories of sociodemographic variables in terms of dimension scores. This may be easily done by Student’s T test and relevant statistical approaches where necessary.

DISCUSSION:

 Further discussions may be added on the added anaylse results (what I suggest above).

Is the work clearly and accurately presented and does it cite the current literature?

Yes

If applicable, is the statistical analysis and its interpretation appropriate?

Yes

Are all the source data underlying the results available to ensure full reproducibility?

Partly

Is the study design appropriate and is the work technically sound?

Yes

Are the conclusions drawn adequately supported by the results?

Yes

Are sufficient details of methods and analysis provided to allow replication by others?

Yes

Reviewer Expertise:

Outcomes Research ; PCH

I confirm that I have read this submission and believe that I have an appropriate level of expertise to confirm that it is of an acceptable scientific standard, however I have significant reservations, as outlined above.

# Reviewer report: Reviewer response for version 1

**DOI:** 10.5256/f1000research.143134.r219042

**Published:** 2023-11-22

**Authors:** Clelia Oliveira Lyra

**Affiliations:** 1 Federal University of Rio Grande do Norte, Natal, State of Rio Grande do Norte, Brazil

The manuscript analyses a valid and reliable quality of life (QOL) assessment tool based on the World Health Organization's Quality of Life Old module (WHOQOL-OLD) because there isn’t translation to Amharic (the widely spoken language in Ethiopia).

 The manuscript is very important for science, especially in Ethiopia and countries that speak Amharic. It is well-written; however, 37/49 references aren't from the last five years. Because of this, it should be to increase the proportion of current literature (2019-2023). The methodology is appropriate, the results are well-described, and the conclusion meets the objectives.

 I suggest you remove the ± symbol, as described in the summary: The results showed a relatively low level of quality of life, with a total transformed score of 58.58±23.15” as they demonstrate the standard deviation. When representing the mean and its respective standard deviation, represent it this way: 58.58 (SD 23.15).

Is the work clearly and accurately presented and does it cite the current literature?

Partly

If applicable, is the statistical analysis and its interpretation appropriate?

Yes

Are all the source data underlying the results available to ensure full reproducibility?

Yes

Is the study design appropriate and is the work technically sound?

Yes

Are the conclusions drawn adequately supported by the results?

Yes

Are sufficient details of methods and analysis provided to allow replication by others?

Yes

Reviewer Expertise:

Epidemiology

I confirm that I have read this submission and believe that I have an appropriate level of expertise to confirm that it is of an acceptable scientific standard, however I have significant reservations, as outlined above.

# Response

**Published:** 2023-11-25

**Authors:** Ahmed Muhye

**Affiliations:** Dire Dawa University, Ethiopia

We appreciate the time and effort that the reviewer has dedicated to providing valuable feedback on our manuscript, "Validation of Quality-of-Life Assessment Tool for Ethiopian Old Age People."

 My co-author and I have revised the manuscript accordingly and would like it to be reconsidered for indexing. 

 Concerning the references, we have tried our best to use the current and in the last five years. However, the problems are: 1) there is a scarcity of research on the validity of tools for quality of life among old age people, and 2) we have done this research from January 16 to March 13, 2021. Hence, the references we used may seem old as we compare them to these years (2023).

 Finally, we are grateful to the reviewers for their insightful comments on our paper. Please let us know if anything further is required at this time, and we thank you very much for considering our revised manuscript.

# Reviewer report: Reviewer response for version 1

**DOI:** 10.5256/f1000research.143134.r208814

**Published:** 2023-11-22

**Authors:** Rozeta Drãghici, Andrada Elena Stan

**Affiliations:** 1 National Institute of Gerontology and Geriatrics "Ana Aslan", Bucharest, Romania; 2 Research Laboratory of Social Gerontology and GerontoPsycholology, National Institute of Gerontology and Geriatrics, Bucharest, Romania

The paper “Validation of Quality of Life assessment tool Ethiopian old age people” by Muhye and Fentahun (2023) was aimed at validating and adapting the World Health Organization Quality of Life for Older Adults questionnaire (WHOQOL-OLD) to the Ethiopian population. The results showed a successful validation of the translated instrument, which was easy to understand and respond to by the participants. The authors were very rigorous with their methodology and statistical analysis by taking into consideration all the necessary aspects. They also provided access to their data, thus making the results reproducible, which is an applauding effort as it promotes the principles of open science.

 The clarity of writing and the structure of the paper represent the aspects that need some tweaking. For example, the introduction is quite short, and it could be improved by presenting and reviewing some papers that have tried to validate and adapt other questionnaires to the Ethiopian population, while explaining why WHOQOL-OLD is a better choice. Moreover, parallels between the prior-used methodology and the one chosen by the authors can be drawn to explain what this paper adds to it. It was noted that there is a section named “Review of existing quality of life assessment tools”, however this section was also incomplete as it did not explain why the other questionnaires do not work as well and how the WHOQOL-OLD overcomes this shortcomings.

 Throughout the paper, there were a couple of mentions regarding nutritionists and their help in this study, but the link between this paper aimed at validating a psychometric tool and the study involving a “mini nutritional assessment” is not clear. It could be of great interest to also focus on nutrition when assessing quality of life, especially when speaking of older adults that have many afflictions that require carefully-designed diets, but this needs to be expanded in writing. Lastly, the results should only be reported once, either in text or in a table, so it is not necessary to have both.

 The paper shows good scientific efforts and validating questionnaires is not an easy endeavour. With some minor improvements, we believe your efforts will be even more obvious to the reader and thus, more appreciated. We wish you the best of luck with your research.

Is the work clearly and accurately presented and does it cite the current literature?

Partly

If applicable, is the statistical analysis and its interpretation appropriate?

Yes

Are all the source data underlying the results available to ensure full reproducibility?

Yes

Is the study design appropriate and is the work technically sound?

Yes

Are the conclusions drawn adequately supported by the results?

Yes

Are sufficient details of methods and analysis provided to allow replication by others?

Yes

Reviewer Expertise:

Gerontopsychology, Social Gerontology, Clinical Psychology

We confirm that we have read this submission and believe that we have an appropriate level of expertise to confirm that it is of an acceptable scientific standard.
